# Telomere Biology—Insights into an Intriguing Phenomenon

**DOI:** 10.3390/cells6020015

**Published:** 2017-06-19

**Authors:** Shriram Venkatesan, Aik Kia Khaw, Manoor Prakash Hande

**Affiliations:** 1 Department of Physiology, Yong Loo Lin School of Medicine, National University of Singapore, 117597 Singapore, Singapore; vshriram87@gmail.com (S.V.); aikkia@gmail.com (A.K.K.); 2 Clinical Research Unit, Khoo Teck Puat Hospital, 768828 Singapore, Singapore; 3 Tembusu College, National University of Singapore, 138598 Singapore, Singapore; 4 VIT University, Vellore 632014, India; 5 Mangalore University, Mangalore 574199, India

**Keywords:** telomeres, telomerase, DNA repair, ageing, cancer, immortalisation, genome instability

## Abstract

Bacteria and viruses possess circular DNA, whereas eukaryotes with typically very large DNA molecules have had to evolve into linear chromosomes to circumvent the problem of supercoiling circular DNA of that size. Consequently, such organisms possess telomeres to cap chromosome ends. Telomeres are essentially tandem repeats of any DNA sequence that are present at the ends of chromosomes. Their biology has been an enigmatic one, involving various molecules interacting dynamically in an evolutionarily well-trimmed fashion. Telomeres range from canonical hexameric repeats in most eukaryotes to unimaginably random retrotransposons, which attach to chromosome ends and reverse-transcribe to DNA in some plants and insects. Telomeres invariably associate with specialised protein complexes that envelop it, also regulating access of the ends to legitimate enzymes involved in telomere metabolism. They also transcribe into repetitive RNA which also seems to be playing significant roles in telomere maintenance. Telomeres thus form the intersection of DNA, protein, and RNA molecules acting in concert to maintain chromosome integrity. Telomere biology is emerging to appear ever more complex than previously envisaged, with the continual discovery of more molecules and interplays at the telomeres. This review also includes a section dedicated to the history of telomere biology, and intends to target the scientific audience new to the field by rendering an understanding of the phenomenon of chromosome end protection at large, with more emphasis on the biology of human telomeres. The review provides an update on the field and mentions the questions that need to be addressed.

## 1. Telomeres–Historical Perspective

In 1938, at a time when even the composition of genetic material was unknown, the existence of a special structure at the ends of chromosomes was first speculated in a lecture given by Hermann Muller [1] that was also validated by Barbara McClintock’s observation of fusion of broken ends of chromatids in meiotic anaphase in *Zea mays* [2]. By 1961, Leonard Hayflick had elegantly demonstrated that cellular ageing is an inherent property of cells—a finding that took 30 years to become accepted [3]. James Watson, in 1972, proposed the ”end replication problem” whereby the 3′ end of the lagging strand of linear DNA is unable to be fully copied during replication due to the removal of the RNA primer from the end, and the inability of DNA polymerase to add nucleotides without a primer [4]. Another independent proposition came from Alexey Olovnikov, a theoretical biologist, who went a step further and proposed that the ends of chromosomes of linear DNA (telomeres) shorten with time (i.e., with every cell division cycle) and that this could be the mechanism behind Hayflick’s observation of cellular ageing in culture [5,6]. However, experimental proof backing the theory and bringing it back into focus would only be obtained two decades later. Elizabeth Blackburn, in 1978, while mapping DNA sequences of the unicellular eukaryotic ciliate, *Tetrahymena*—which tends to have thousands of mini-chromosomes upon differentiation—observed one particular repeat sequence (TTGGGG)_n_, at the ends of the chromosomes [7]. Those sequences, which were able to rescue linear DNA, introduced into yeast, from degradation by nucleases, were dubbed ‘telomeres’. Dramatically, the same observation in yeast by Szostak and Blackburn, upon introduction of telomere-containing linear DNA from *Tetrahymena*, a completely unrelated species, also highlighted the maintenance of chromosomal integrity by telomeres as a fundamental cellular mechanism [8]. This would soon lead to the unravelling of a distinct pattern conserved across all eukaryotes—a guanosine-rich hexameric DNA repeat sequence at the chromosome ends which, in humans, was later revealed to be composed of 5′-TTAGGG-3′ repeats [9,10]. In 1985, counterintuitive to the prevailing speculation that telomere shortening happened with cellular ageing, a member of Blackburn’s lab, Carol Greider, observed that *Tetrahymena* telomeres were getting longer with every generation during its logarithmic phase of growth; leading to the discovery of an enzyme, telomerase, with terminal transferase activity, for which she would go on share the 2009 Nobel Prize with her mentor Blackburn, and Szostak [11]. The first experimental hints of telomere shortening, per se, were obtained from the finding that telomere lengths varied considerably among different tissues [12]. Direct evidence of telomere shortening with increasing passage number followed soon after by studies on cultured human fibroblasts in vitro and primary human skin cells [13,14]. Discoveries in the field started to happen at a fervid pace in the 1990s. Further investigations on identifying telomerase activity in various human tissues revealed significant activity in germline cells and cancer cells [15,16]. While the core components of human telomerase holoenzyme—human telomerase RNA (hTR), and a catalytic subunit, human telomerase reverse transcriptase (hTERT)—were being identified, signs of existence of an alternative telomere-lengthening mechanism in humans was being observed as well [17,18,19,20]. Another landmark study found that the introduction of hTERT into normal human cells led to the extension of their lifespan by at least 20 population doublings [21]. This study formed the basis of a tantalising link between telomeres and senescence (irreversible cell cycle exit). A rather obscure field until then, telomere biology, with a cornucopia of finer details, replete with surprising connections, astounding counter-intuitions, and promising implications to medicine and ageing, has catapulted itself to the forefront of science in the last two decades. We discuss below all of the fundamental aspects of telomeres, beginning with the bare bones—the telomere sequence.

## 2. Telomere Sequence

Telomeres are composed of hexameric tandem repeats with each repeat containing three or more guanosine residues. Vertebrates, regardless of chromosome number or length, have highly-conserved 5′-TTAGGG-3′ terminal repeats with a long double-stranded region (ranging between 5 and 50 kb across species) and a short single-stranded overhang or the G-tail (ranging between 100 and 300 nt across species) [9,22,23,24]. The telomeric repeats protect chromosome integrity while buffering the loss of terminal DNA due to the inherent ‘end-replication’ problem. Strikingly enough, cells largely suffer with mutated telomeres, exhibiting rampant chromosome segregation errors, a checkpoint distinct from typical DNA damage (irrespective of the telomere length), and an altered growth phenotype in yeast [25]. It is unclear as to how the cell perceives these mutations.

## 3. Telomere-Associated Protein Complexes and Other Accessory Factors

Certain protein complexes and other players associate with, and expose, telomeres to legitimate interacting partners, like telomerase, for instance, during telomere replication in the S phase, while protecting them from factors that trigger persistent DNA damage response (DDR) and illegitimate recombination by the MRN complex. Though not all is known of their interactions in various cellular states, the past decade has seen the unravelling of many new and unexpected factors associated with telomeres.

### 3.1. The Shelterin Complex, Telomeric Structure, and the Telomeric DNA Damage Response

A complex of six proteins that binds to telomeres has been specifically identified and named ‘shelterin’. The subunits in mammals—TRF1, TRF2, TIN2, RAP1, TPP1, and POT1—were identified by looking for proteins with binding specificity to telomeric repeats using sequence homology (with their unicellular counterparts) and by searching for protein-protein interactions at telomeres within a span of 10 years [26,27,28,29,30,31,32,33]. TRF1, TRF2, and POT1 bind to telomeric DNA with exquisite specificity [29,34,35,36,37]. POT1 binds to the single-stranded G-overhang and to the single-stranded region of a telomeric secondary structure, elaborated below [27,36,38,39]. While TIN2 tethers TRF1 and TRF2, TPP1 tethers POT1 to TIN2. At the outset, telomere-binding protein complexes in evolutionarily-distant species like *Saccharomyces cerevisiae*, *Oxytricha nova*, *Schizosaccharomyces pombe*, and mammals, seem to vary considerably in the number and homology of proteins. Evolutionary conservation of the shelterin-like roles of these proteins seem to occur at the level of the modular domains that enable their recognition of and binding to single-strand overhangs, double-stranded DNA, and telomere-sequence recognition, reviewed in detail elsewhere [40].

**Figure 1:**
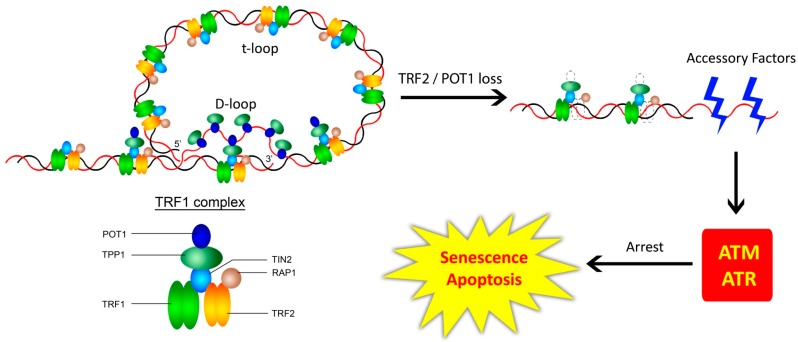
Shelterin—The Border Security Force. The longstanding basic model posits that the six protein-complex directly associated with telomeres facilitates loop formation and protects it from illegitimate access by various factors, regulates access by the legitimate ones, and aids telomere replication. ‘t-loop’ stands for telomeric loop, while ‘D-loop’ stands for displacement loop. In mammalian cells, loss of shelterin proteins can lead to DDR, TRF2, and POT1 directly serving to inhibit ATM and ATR kinases, respectively, and a loss of TRF2 or POT1 would de-repress the association of ATM/ATR to the telomeres, resulting in senescence or apoptosis. Adapted from [42].

One issue that the telomere structure (dsDNA region followed by an ssDNA overhang) could face is the recognition of the G-tail or ssDNA overhang as a canonical DNA break by DDR factors. Analysis of human telomeric DNA revealed that the shelterin complex, especially TRF2, aids telomeres in attaining a closed configuration in the form of a loop, called the ‘t-loop’, or the telomeric loop [41]. TRF2 and POT1 serve to protect the double-stranded and single-stranded regions of telomeric DNA by preventing the activation of ATM and ATR kinases, respectively, while the t-loop seems to prevent access of DNA damage sensors with DNA-end binding ability to the telomeres (Figure 1) [42]. While even inhibition of TPP1 causes ATR activation, it is because of POT1 being unable to tether to the TRF1/2 node of the shelterin to repress ATR [42]. The G-tail is also crucial to permit dynamic interactions with various players outlined below and integral to the functionality of the telomeres. Apart from the t-loop, telomeres are also prone to forming more secondary structures like the G-quadruplex (reviewed elsewhere) which, by themselves, have been implicated in regulatory roles in the genome [43]. Telomeres pose an ‘end replication problem’, not only due to the loss of lagging telomeres during replication, but also due to the complete replication at leading telomeres, resulting in blunt ends. While the presence of a G-tail could be considered an inevitability of the lagging strand being incompletely replicated, it is striking that the cell processes even the blunt telomere after replication at the leading strand to create an overhang. First brought to light in 1993, a simple answer to the phenomenon still eludes us [44]. Thus, evading common perception—the recurrent theme of telomere behaviour—the overhang might actually render the telomeres the ability to be capped and protected [45,46].

Understanding the biology of the shelterin complex is pivotal to understanding telomere functionality. Deregulation of the components of the shelterin complex results in chromosomal instability and changes in telomere length [32,39,47,48]. More detail on shelterins is dealt with elsewhere in other reviews [36].

The fastest shaping problem in telomere biology is the modulation of DDR by the shelterin complex. While the prevailing notion is that shelterin serves to dampen DDR at the telomeres, it fails to explain the longevity of functional telomeres despite accumulating irreparable damage over time [35]. A recent study found evidence of DNA compaction at the telomeres by shelterin and claimed that the modus operandi of shelterin was to keep out DDR proteins and that compaction was a great way of doing so [49]. Two studies have just been published which, together, show that compaction happens only in a fraction of telomeres and that the telomeres were completely functional in the absence of compaction [50,51]. They also add that the compaction model could possibly not explain how a very large accessory complex, such as the MRN complex, could access the telomeres without trouble [50].

The other rapidly-evolving question is the nature of DNA repair at the telomeres. It has been established that a repair signal at the telomeres is detrimental to its survival. Studies in senescent cells reveal that telomeres mounting DNA damage-based checkpoint activation seem to be the main trigger behind a senescence programme [52,53]. Even a single DSB at the sub-telomeric region of mouse embryonic stem cells had a great effect on chromosomal instability [54]. Damage to interstitial DNA is usually completely repairable, but sub-telomeric and telomeric DSBs have been observed to be refractory to repair and elicit persistent DDR [55,56,57]. However, the characterisation of DSBs as telomeric was based on damage induction by ionising radiation and visualising TRF2 foci as marker for telomeres—both being indirect ways of getting at the problem [56]. A recent study induced DSBs specifically within the telomere tract using a telomere-directed endonuclease and observed DNA repair activation [58]. They show that shelterin, in fact, does not repress DDR activation and, thus, repair happens by ATM-mediated HR and PARP1-mediated end joining. Thus, the notion about damaged telomeres mounting a unique DDR profile as compared with a damaged interstitial DNA segment, and their persistence, just gained more nuance. While telomeric damage seems to be promptly recognised and repaired, it is the damage in the vicinity of the telomeres that is refractory to repair and mounts a constitutive DDR signal, whereas the cells are well-evolved to repairing damage to interstitial DNA robustly. It is fascinating to imagine how fine-tuned these responses are and the importance of maintaining the integrity of telomeres.

### 3.2. DNA Repair Factors—Friend or Foe to the Telomeres?

Telomeres exhibit yet another behaviour that is counterintuitive to the common notion that associating with, and activating, DNA repair factors is detrimental to cell survival. An early observation that formally tethered efficient telomere maintenance with the presence of intact DDR factors included an apparent telomere dysfunction phenotype in cells from Ataxia telangiectasia (AT) patients [59]. In a study, the first of its kind, cells from mice that lacked ATM exhibited accelerated telomere shortening, accumulation of extra chromosomal telomeres and chromosome alterations [60]. Eventually, ATM, PARP1, DNA-PKcs, Ku70/80, and XRCC4, among others, were all implicated in the maintenance of telomere function, in addition to their role in DNA repair [61,62,63,64,65,66].The MRN complex, known to bind to DNA and activate an ATM-mediated DDR, has been shown to associate with TRF2 at the telomeres [67]. The execution of an NHEJ response near the telomeres is controlled greatly by ATM kinase, unlike that in interstitial DNA where ATM regulates NHEJ to a lesser extent; and a failure to activate NHEJ at the telomeres and sub-telomeric regions results in large deletions and gene rearrangements, leading to catastrophic chromosomal instability in human cells [56,57,68]. The most intimate relationship of DDR factors to telomere maintenance has been recently shown in two studies in both human and mouse cells—that ATM and ATR kinases are necessary for telomere elongation [69,70].

One possible reason for this intriguing relationship could be that it may allow for efficient control over cell cycle progression [71]. Moreover, at least some of the DDR proteins implicated at the telomeres have some novel function, independent of their checkpoint activation and DDR functions [72]. It is also possible that the stoichiometry of DDR proteins to the shelterin complex determines the balance between telomere protection and initiation of unrestrained damage response. Additionally, the observation that there is no striking structural commonality among all of the DDR proteins in telomere maintenance points to the possibility that telomere maintenance is not a discrete function of the cell’s machinery, but one that is integrated with DNA maintenance [73].

Nevertheless, targeting these DDR factors in the purview of cancer therapy may serve to dampen canonical DDR as well as weaken telomere protection, even in cancers with stable genomes. Thus, despite the lack of a sophisticated understanding behind the intertwined fates of DDR factors and telomeres, targeting telomere maintenance and DNA repair in cancer cells has been a strategy much resorted to in the past decade of research.

### 3.3. New Kids on the Telomere Block

The shelterin complex has been the most well-characterised of telomere-binding proteins for their telomere-guarding function and has been the most deterministically implicated one in human diseases by virtue of telomere dysfunction due to defects in one or more of the shelterin proteins. While shelterins have been the focus of the review, it is worth addressing some emerging and new players at the telomeres.

#### 3.3.1. CST (CTC-STN1-TEN1)

One discovery of the past decade based on the approach of looking for human homologues of protein complexes found in yeast was that of the CST complex at the telomere [74,75]. CST complex, consisting of CTC1, STN1, and TEN1, acts independently of the shelterin complex and ensures smooth replication at telomeres and replication restart after stalling of the replication fork [76], while reportedly ensuring that telomerase acts only once per cell cycle on each telomere [77]. STN1 is required by DNA polymerase-α for complete extension of the telomere following telomerase action [78]. CST complex, although shown to bind to telomeres independently of POT1, has been surmised to interact with shelterin to protect telomeres [74,79].

#### 3.3.2. HOT1 (Homeobox Telomere-Binding Protein 1)

Active telomerase is proposed to be assembled in Cajal bodies in the nucleus by bringing hTERT and hTR together [80]. While the complete mechanism by which telomeres are recruited to the proximity of telomerase in the Cajal bodies is not well understood, HOT1, a newly-identified protein that binds directly to telomeres, seems to aid in localising telomere sequences to Cajal bodies that contain TERT [81,82].

### 3.4. Telomeric RNA

#### 3.4.1. TERRA (Telomere Repeat-Containing RNA)

Contrary to prior notions, telomeres are indeed transcriptionally-active regions, giving rise to long non-coding RNA called TERRA [83]. TERRA levels are tightly regulated through the cell cycle as TERRA affects the replication of leading-strand telomeres [84]. TERRA has also been found to orchestrate the binding of POT1 and RPA to the telomere ssDNA and ensures that POT1 displaces RPA promptly after replication is complete as RPA is known to activate DDR [85]. Thus, TERRA seems to play an important role too, in regulating the telomere capping state, depending on the cellular context. A recent study has shown severe loss of telomeres and a dramatic DDR when the TERRA locus was deleted by CRISPR, underscoring the necessity of TERRA to maintain telomeres [86]. An elegant study probing the effects of gene silencing by intact, long telomeres, ended up finding that, in cancer cells, TERRA was increasingly produced by long telomeres, and that this enabled silencing of some innate immune genes and in turn promoted their cell differentiation in vivo [87]. Specifically, this activity of TERRA was nailed down to its ability to form G-quadruplex stretches [87]. This renders TERRA a highly attractive cancer drug target.

#### 3.4.2. tDDRNA (Telomeric DNA Damage Response RNA)

Following the discovery of site-specific small non-coding RNAs in modulating DNA repair response (DDRNAs) [88], telomere-specific DDRNAs have been very recently reported and has been claimed to be required to mount a DDR at the telomeres [89]. RNAs certainly have a significant part to play in orchestrating the breathtaking complexity of linear DNA end-protection and unravelling their roles is set to be the next frontier in telomere biology.

## 4. Telomere Length Maintenance 

Telomeres are a solution to the problem of maintaining biologically-functional linear DNA. Perturbation of the telomere structure unleashes a host of negative responses leading to cell arrest or even cell death. The cell processes telomeres scrupulously—telomere replication, telomere trimming, and telomere elongation are all part of this. Telomere stability in stem cells has recently been reported to be a result of a dynamic balance elongation and trimming mechanisms, some of which have been outlined in the study [90]. A very recent study also discovered a specific protein, TZAP, that directly competes with shelterin for telomere-binding and facilitates trimming of ‘excessively long telomeres’ [91]. While telomere replication has been reviewed elsewhere [92] and telomere trimming is a highly nascent area of study, this review focusses on telomere elongation mechanisms—the implications of which profoundly impact medicine and ageing.

### 4.1. Telomerase: What Is True of the Ciliates Is True of the Elephant Too!

One mechanism of telomere elongation that is common across evolutionarily-unrelated species is that by the activity of telomerase. Telomerase is a ribonucleoprotein (RNP), that is comprised of the catalytic subunit (hTERT in humans) and the telomerase RNA (hTR in humans), apart from a host of other proteins. The assembly of the holoenzyme has been observed to be a complex process, involving multiple components over multiple locations in the nucleus [93]. Overall, the prevailing notion was that active telomerase, when assembled in Cajal bodies (which has well-known RNP assembly functions), carries out de novo telomeric repeat addition with great processivity (acting on the same telomere again and again) and activity (acting on several molecules of telomeres after processive addition on one molecule) [94,95]. The role of Cajal bodies in the assembly of telomerase has recently been challenged by two elegant studies—both of which show that telomerase activity and telomere maintenance are not compromised in human stem cells and cancer cells lacking coilin and, hence, those that cannot form Cajal bodies [96,97]. Vogan et al. also go on to demonstrate the minimization of telomerase to functional units, stripping down most other components, thereby opening key insights into the role of the complex telomerase biogenesis pathway [97]. Solving the telomerase structure would give us invaluable detail on the telomerase reaction cycle, and the intricate dynamics, and is reviewed elsewhere [98].

Normal somatic cells in humans lack telomerase activity. Access to telomeres and the extent of elongation by telomerase is tightly regulated by a sophisticated network of telomere-associated proteins. Consequently, approximately 90% of the cases, those clones that reactivate the otherwise repressed telomerase go on to become cancer cells [16,99]. The timing of telomerase activation in cancers is varied and, thus, postulated to be highly dependent on the tissue of origin, and the microenvironment [100]. Telomerase reactivation has been shown to happen in a majority of cases by overexpression due to chromosomal rearrangement to be juxtaposed to a highly active promoter, or alternative splicing of hTERT, which codes for the catalytically-active protein subunit telomerase reverse transcriptase [100]. Mutations to the TERT promoter is also a powerful way to reactivate TERT robustly, potentially driving carcinogenesis [101,102]. While the intricate network of telomerase regulation that would lead to its reactivation is still a vaguely understood niche [103], any breakthrough into its fundamental working will have a significant impact in cancer treatments.

### 4.2. Alternative Lengthening of Telomeres (ALT)

The first glimpse of cells’ ability to survive and divide without telomerase was seen by Blackburn in 1993, who reported the stunning observation of cells with a telomerase-independent maintenance of telomeres, leading to cell immortalization [104]. Soon, similar observations of prolonged survival of several mammalian cell types in a telomerase-null background was reported by a few pioneering studies [105,106,107]. Since then, a body of research has shown that most of the 10–15% of cancers that are telomerase-negative seem to maintain their telomeres by ALT mechanisms, broadly by recombination between telomeres of different chromosomes. ALT activity has been observed more frequently in cancers of mesenchymal origin, namely glioblastoma multiforme and osteosarcoma [108,109].

The proteins of the HR pathway of DNA repair known to facilitate inter-chromosomal telomeric recombination in ALT are present in normal cells as well, where they perform normal DNA recombination and repair functions in response to DNA damage [108]. While the mechanism that helps normal cells prevent those proteins from engaging in ALT-associated telomere recombination is not precisely known, the persistence of telomeric DDR and the absence of chromosome fusions in ALT cells calls for the abundance of telomeres that are not in the native conformation, yet not entirely dysfunctional [110]. An altered chromatin state by means of the loss of ATRX (a chromatin remodelling protein), and decompaction of the telomeres, have been implicated by some recent studies [111,112,113]. Indeed, a set of elegant studies has recently shown that DNA damage even at ALT-negative telomeres stimulates telomere movement; in ALT cells, resulted in telomere clustering in characteristic APBs (ALT-associated PML bodies, biomarker for ALT activity) [110,114,115]. Strikingly, Rad51 tracked the path of telomere movement accurately, and its inhibition led to a suppression of the phenotype [116]. Thus, ALT telomeres, owing to the activation of a DDR, harness the ability of Rad51 filaments to aid in a homology search across long distances and maintain functionality.

The interplay between ALT and telomerase-mediated elongation, and their effect on telomere homeostasis is yet to be brought to light, although there have been observations of their coexistence in normal mouse somatic cells and human tumour samples recently [117,118,119]. Emerging evidence points to the localisation of telomeres as a compelling drive in determining the mechanism of maintenance. Telomeres of ALT-positive cells have interspersed variant repeats throughout the telomeres, enabling the modification of the telomeric chromatin, including binding by nuclear receptors [120]. Telomerase elongation requires the assembly of active telomerase holoenzyme, and the localisation of telomeres at Cajal bodies. It is, thus, possible that while recruitment of nuclear receptors to telomeres may facilitate recombination by oligomerisation of telomeres, it may also prevent their localisation at Cajal bodies for elongation by telomerase [80,120]. Nonetheless, it stands out that much of the underlying mechanisms remains to be unravelled in order to exploit telomere-maintenance mechanisms for cancer therapy.

## 5. Telomeres and Cell Physiology

### 5.1. Telomeres and Cell Turnover

**Figure 2:**
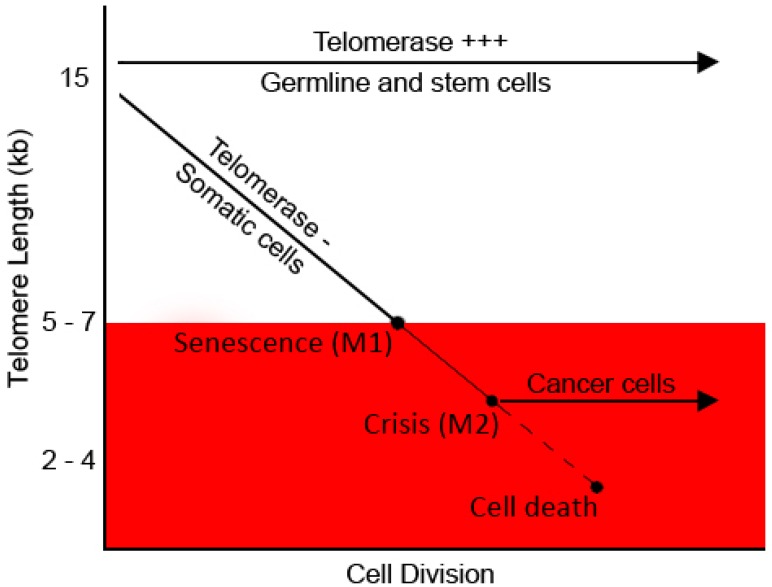
Telomere length and cellular ageing. Telomere shortening-mediated cell arrest occurs in somatic cells proliferating for a few cycles (M1). Non-arrested cells undergo progressive telomere shortening and die by apoptosis at M2. Cells that have a mutated apoptotic checkpoint encounter death due to massive genomic instability. Modified from [123].

Telomeres protect chromosome integrity and, hence, genome stability to a great extent. Telomeres are deemed dysfunctional if either their telomere length has reached a critical set-off point or the associated shelterin units are delocalised from the telomeres. Telomeres shorten with every cell replication: mitotic cells are consequently more prone to genetic instability as opposed to non-dividing cells. Conceivably, multicellular eukaryotes have evolved mechanisms to signal senescence in mitotic cells upon encountering such stresses. This form of telomere-dependent replicative senescence is characterised by an irreversible exit from cell cycle, resistance to apoptotic signals, and a grossly altered gene expression profile. Telomere shortening, thus, underlies an elegant method adopted by *Homo sapiens* for maintaining cellular homeostasis and cell turnover (Figure 2). Non-dividing cells undergo telomere-independent senescence upon encountering other kinds of stresses like a sudden mitogenic trigger. Although senescence clearly serves as a barrier for carcinogenesis [52] in exceptional cases, telomere shortening beyond the critical point could predispose cells to acquiring a “mutator phenotype” [121,122].

### 5.2. Telomere Shortening in Stress and Ageing

Diseases driven by defects numerous telomere-associated factors and telomere maintenance are collectively called telomeropathies and have been extensively reviewed elsewhere [124,125,126,127]. It has been well established that telomere shortening limits cellular lifespan. Over the years, the notion that telomere length governs mammalian lifespan has emerged. However, this is an oversimplification of telomere biology, and even exaggeration of its role in ageing. Organismal ageing is governed by not just a few, but a multitude of factors.

Telomere length, interestingly, has been shown to be affected by stress per se by mechanisms that are not very well characterised. Psychological health, especially until early adulthood, and lifestyle have been shown to influence telomere length [128]. Extremely short telomeres lead to the causation of telomere syndromes, such as dyskeratosis congenita, causing accelerated ageing [125]. Amidst widespread misconception that telomere shortening directly correlates with the mammalian lifespan, a pioneering study in mice has shown that the rate of increase of critically-short telomeres determines ageing, rather than the mean telomere length [129]. Leukocyte telomere length has been shown to be a predictor of cardiovascular ageing [130]. Despite the obscure underlying mechanisms, telomere length is an informative biomarker of mammalian ageing, and also of a wide spectrum of diseases which affect a significant population.

While telomere length association studies in numerous contexts have stolen the limelight lately, it is essential for us to understand the underlying mechanisms deeply, in order to explain any departure from the correlation or even the lack of correlation of telomere length with any given parameter of assessment. An interesting study, off the beaten path, observed chromosome looping, enabling telomeres to access distant genetic loci in their respective chromosomes and affect their silencing [131]. Indeed, while this looping was observed in cells with long telomeres, it was abrogated in isogenic cells engineered to possess short telomeres, thus laying the foundation for what the authors named TPE-OLD (telomere position effect over long distances) [131]. Taking note of this, Theodoris et al. studied an age-dependent cardiac disease caused by haploinsufficiency of NOTCH1 [132]. Using mice lacking telomerase RNA (and, hence, undergo telomere shortening, like human somatic cells), and heterozygous for NOTCH1 (and, hence, haploinsufficient), upon validating their model to undergo age-dependent telomere shortening they show that the severity of the disease correlated with telomere length. Examining gene expression alterations over a few generations, they were able to correlate a change in the expression pattern with telomere shortening, and also by using 3D chromatin analysis, showed that the promoters of those genes specifically were contacted by telomeres [132]. The study, a classic demonstration of successful clinical application of such a basic finding with respect to telomeres, will hopefully serve as a bellwether for more studies on various diseases.

### 5.3. Telomeres and Cancer–Therapeutic Approaches

**Figure 3:**
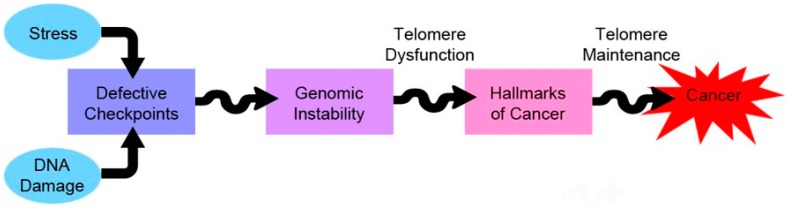
Telomeres and cancer—a love/hate relationship. Telomere dysfunction precipitates the acquisition of other hallmarks of cancer by increasing the rate of chromosomal instability. However, it also poses a threat of induction of cell death during mitosis, due to massive genomic instability—named mitotic catastrophe.

Cells with telomere dysfunction, when left unchecked, can spiral towards a genetically-unstable phenotype by way of chromosomal instability. This precipitates the acquisition of cancer hallmarks, provided the cells are able to regain the ability to maintain their telomeres. Ironically enough, telomere maintenance is necessary for the emerging cancer clones to become full-blown cancers (Figure 3) [133].

There is evidence that cancer cells that maintain their telomeres by either mechanism—telomerase or ALT—are characterised by telomeres interspersed with variant repeats, although to varying extents [134]. Cancer cells, thus, maintain their telomeres more vigorously, and in a way that is different from normal somatic cells. Targeting telomere homeostasis has been a promising strategy in cancer therapy as normal cells would presumably take more severe exposure to become affected, as compared to cancer cells.

Telomerase activity has been shown to be a diagnostic and prognostic marker for a wide range of cancers [135,136,137,138]. Thus, anti-telomerase treatment strategies have been actively researched and would hopefully improve survival rates of cancers in the coming years. While the search for more desirable drug candidates is on, our laboratory has, in the past, characterised some natural plant products for their anti-telomerase activity in breast and brain cancer cell types [139,140,141,142,143]. While telomerase-positive cancers have been subject to the majority of preclinical studies and clinical trials [144], developing effective treatments against ALT-positive cancers is crucial for two reasons—ALT occurs with overwhelming majority of some classes of solid tumours which have extremely poor prognosis, and telomerase-positive cancers can switch to an ALT-positive one either sporadically or due to selection imposed inadvertently by therapeutic interventions [145,146]. Remarkably, ALT cancers were shown to be hypersensitive to ATR inhibition: Flynn et al., by exploiting the fact that ALT cancers lack ATRX (the chromatin remodeller), showed the inability of TERRA to displace RPA from the telomeres following replication and, hence, to silence ATR activation [147]. The use of ATR inhibitors proved lethal for the ALT-positive osteosarcoma cells [147]. This study serves as a beacon for ALT-based preclinical investigations and hopefully more studies will follow suit.

## 6. Concluding Remarks

Telomeres are one of the main guardians of genome stability, and the last two decades of research has seen the realisation of genomic instability as a main underlying event during tumorigenesis in an overwhelming majority of cases. Although the role of telomeres in cancer cell physiology has been evident for a few years now, the biology of telomeres is only now being studied in unprecedented detail. Moreover, the role of telomeres in central themes like stress and ageing, is also being unravelled at a feverish pace, and this augurs well for smarter therapeutics in the near future, not just for cancers, but for a much wider spectrum of complex diseases. With the questions highlighted in this review being answered with an ever-expanding group of scientists, armed with an unprecedented range and power of techniques to probe into the cell, the next decade is surely to bring about a slew of watershed discoveries in this field.
